# Assessing the clinical reliability of short-term heart rate variability: insights from controlled dual-environment and dual-position measurements

**DOI:** 10.1038/s41598-025-89892-3

**Published:** 2025-02-15

**Authors:** C. Besson, A. L. Baggish, P. Monteventi, L. Schmitt, F. Stucky, V. Gremeaux

**Affiliations:** 1 Sports and Exercise Medicine Center, Swiss Olympic Medical Center, Lausanne University Hospital, Lausanne, Switzerland; 2 Institute of Sports Sciences, University of Lausanne, Lausanne, Switzerland; 3 Department of Cardiology, Lausanne University Hospital, Lausanne, Switzerland; 4 Cardiovascular Performance Program, Division of Cardiology, Massachusetts General Hospital, Boston, Massachusetts USA; 5 Faculty of Biology and Medicine, University of Lausanne, Lausanne, Switzerland; 6 National School of Mountain Sports/National Ski-Nordic Centre, Premanon, France; 7 College of Sports Science and Technology, Mahidol University, Bangkok, Thailand

**Keywords:** Heart rate monitoring, Heart rate control, Autonomic nervous system, Statistics, Reproducibility of results, Physiology, Cardiology

## Abstract

Heart rate variability (HRV) is a widely recognized biomarker for autonomic nervous system regulation, applicable in clinical and athletic settings to monitor health and recovery. Despite its extensive use, HRV measurement reliability is influenced by numerous factors, necessitating controlled conditions for accurate assessments. This study investigates the reliability of short-term HRV measurements in various settings and positions, aiming to establish consistent protocols for HRV monitoring and interpretation. We assessed morning HRV in 34 healthy, physically active adults across supine and standing positions, at home and in the laboratory, over a 24-hour period. Environment significantly impacted standing HRV. Home measurements exhibited slightly lower variance compared to lab settings, underscoring the importance of environment control. Our findings confirm the high reliability of HRV measurements, indicating their robustness in capturing autonomic changes, provided a rigorous methodology is employed. Here we show that effective and reliable HRV assessment is possible across various conditions, contingent upon strict management of confounding factors. This research supports the utility of HRV as a non-invasive diagnostic tool, emphasizing its importance in health management and potential in broadening applications to diverse populations. Future studies are encouraged to expand these assessments to include varied demographic and clinical profiles, enhancing HRV integration into routine health evaluations.

**Supplementary Information:**

The online version contains supplementary material available at 10.1038/s41598-025-89892-3.

## Introduction

Heart rate variability (HRV) – the fluctuation in the time intervals between consecutive heartbeats – is an indirect measure of autonomic nervous system (ANS) regulation. Its significance in both clinical and non-clinical areas has grown considerably since its early application in fetal monitoring and post-myocardial infarction care in the 70s and 80s^1,2^. Since then, HRV has been explored in diverse areas from mental health and sleep disorders to sports performance, as it provides a valuable window into an individual’s physiological and psychological states, including stress levels, recovery capacity, and overall health. In clinical settings, it serves as a sensitive biomarker in conditions such as sleep disorders^3,4^ and stress-related illnesses, depression, and anxiety^5,6^. Outside of strictly clinical applications, HRV has gained traction as a tool for monitoring training load, recovery, and readiness in athletes^7,8^. Recent research further underscores HRV’s pivotal role in capturing the dynamic interplay between the brain and heart during cognitive and emotional processes: error-related cardiac deceleration, or the transient slowing of the heart following an error, illuminates the tight coupling between performance monitoring and autonomic reactivity^9^, while fear-induced bradycardia has emerged as a psychophysiological measure of defensive responding in fear conditioning paradigms, offering fresh insight into stress-related and psychopathological conditions^10,11^. Together, these findings suggest that HRV—and more broadly cardiac variability—transcends a simple risk marker, instead serving as a key index of brain–body integration. Disruptions in this integration may underlie various psychiatric, physiologic and neurological disorders^12,13^.

Despite its wide utility, HRV analysis is not without challenges. One significant obstacle is that HRV can vary considerably depending on a host of psychological, physiological, methodological, and environmental factors. Over the past few decades, researchers have attempted to establish consensus guidelines for HRV data collection and interpretation—most notably through the foundational Task Force report in 1996 and more recently by Quigley and al. —but the field still lacks a universally accepted standard^14,15^. For instance, there remains debate over issues like the optimal timing of data collection (e.g., upon awakening, during nighttime, or pre/post-exercise or intervention), the duration of recordings (e.g. 5-minute “short-term” vs. 24-hour monitoring), and the body position to be used (e.g. supine, standing, or seated)^15–17^. Each of these factors can influence HRV metrics, leading to inconsistencies and difficulties in comparing results across studies^18–21^. Hence, reliable and feasible protocols are urgently needed to enhance the robustness and reliability of HRV research.

One promising strategy is the use of short-term HRV measures – often ≤ 5 min – that balance practical feasibility with sufficient diagnostic and research value^22^. A shorter recording period reduces participant burden, is easier to replicate in both clinical and athletic contexts, and may be less prone to artifacts introduced by movement or posture changes^14,23^. As portable technologies advance, short-term HRV measurements have become increasingly accessible, allowing assessments to be performed even in non-traditional settings such as homes, workplaces, or sports field. However, while short-term assessments are convenient, they still necessitate careful consideration of methodological variables to ensure consistency.

In this regard, a dual-position HRV protocol has gained particular attention^24,25^. This approach captures HRV in both supine and standing positions to account for the physiological challenges associated with each posture^26^. In the supine position, the cardiovascular system experiences minimal gravitational stress, generally resulting in higher parasympathetic activity. Conversely, standing requires orthostatic adaptation and typically increases sympathetic outflow to maintain blood pressure^26^. Monitoring HRV in both positions thus provides a more comprehensive snapshot of ANS function, capturing a balance between parasympathetic and sympathetic responses. Studies have suggested that this dual-position method is particularly useful for differentiating types of fatigue, as it allows researchers to observe changes in low-frequency (LF) and high-frequency (HF) bands under two contrasting autonomic loads^24,27,28^. If administered with rigorous standardization—ideally at consistent times, such as upon awakening—the dual-position protocol has the potential to yield reliable and context-rich data that inform tailored interventions in both healthcare and sport.

Nevertheless, questions remain about the stability and generalizability of these measurements across different environments and populations. Laboratory-based assessments offer controlled conditions, but the resulting data may not fully reflect an individual’s daily life, particularly when measurement timing varies^29^. Conversely, home-based assessments provide ecological validity and convenience, but introduce potential uncontrolled variables, such as room temperature. Additional factors including participant familiarity with the measuring device and adherence to pre-measurement guidelines (e.g., abstaining from caffeine) can further influence results^12,30,31^. These considerations highlight the importance of reliability studies designed to examine how HRV measures vary under different conditions and over time, particularly when applying the dual-position approach.

Against this backdrop, the present study aims to address a gap in the literature regarding the reliability and interpretative value of a standardized morning dual-position HRV protocol. Building on recent efforts to streamline short-term HRV assessments, our work investigates the performance of these measurements in healthy, physically active individuals across both at-home and laboratory settings. We hypothesize that this dual-position protocol will provide reliable indicators of autonomic function, regardless of the data collection environment, while also revealing trivial day-to-day variability. Specifically, our study focused on four main objectives: (1) assessing overall short-term HRV reliability over a 24-hour period; (2) analyzing day-to-day variance; (3) comparing at-home and in-laboratory measurements performed on the same day; and (4) evaluating short-term variance in lab conditions. By measuring HRV across multiple time points and settings, we aimed to assess whether the protocol reliably captures shifts in HRV between supine and standing positions, while accounting for potential effects of environmental stressors and participant comfort. If proven valid and reliable, this dual-position method could guide individualized interventions in healthcare and sports, enabling clinicians to track cardiac rehabilitation progress and coaches to optimize training and recovery. Moreover, establishing HRV reliability in a young, active population also establishes a basis the way for future investigations in clinical cohorts, with impaired autonomic regulation. This research aims to examine the reliability and applicability of this method in realistic scenarios. The findings will contribute to an evolving dialogue on HRV measurement best practices, potentially paving the way for broader adoption of dual-position assessments in routine clinical practice and athletic monitoring.

## Results

### Participants

**Fig. 1:**
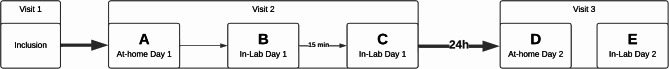
Study design.

Out of the initial 43 participants recruited for this study, 34 successfully completed five HRV measurements (measure A, B, C, D, E) under the designated conditions and were included in the final analysis. Nine participants were excluded due to non-compliance with the experimental protocol, incomplete measurements, symptomatic responses (orthostatism), or technical issues that affected data integrity. Additionally, three standing measurements from the included 34 participants were excluded due to similar reasons. Detailed justifications for these exclusions are provided in the supplementary materials. Figure 1 presents the study design. All measurements were conducted during two consecutive mornings, both at home and in the laboratory, with an average interval of 1h01 ± 15 min on the first day and 1h03 ± 19 min on the second day. Table 1 outlines the characteristics of the participants and the timing of the measurements. Importantly, none of the participants exhibited significant fatigue scores on the Profile of Mood States (POMS) questionnaire, ensuring the homogeneity of the fatigue state of the population studied.

**Table 1:** Gender repartition, age, weight, and height of participants with POMS results and time of measurement. Data are presented as mean ± SD. TMD, total mood disturbance; POMS, profile of mood state.

M/F	19/15
Weight (kg)	67.8 ± 12.5
Height (cm)	173.9 ± 8.6
BMI (kg/m^2^)	22.3 ± 3.1
Age (y)	30.3 ± 9.3
Training weekly hours	4.6 ± 3.3
Training weekly sessions	3.1 ± 1.7
Sport type	Badminton (1); Basketball (2); Biking (3); Dancing (1); Football (2); Gym (2); Hiking (1); Ice Hockey (1); Martial arts (1); Multisport (5); Running (8); Squash (1); Swimming (1); Tennis (1); Triathlon (1); Unknown (1); Volleyball (1); Yoga (1)
Tension	9.8 ± 4.1
Anger	8.4 ± 8.3
Fatigue	5.4 ± 4
Vigor	18.5 ± 4.8
Depression	5.9 ± 8.4
Confusion	7.0 ± 3.2
TMD	18.0 ± 24.3
Measure A	6h44 ± 51 min
Measure B	7h46 ± 50 min
Measure C	8h11 ± 50 min
Measure D	6h49 ± 51 min
Measure E	7h53 ± 48 min

### Descriptive statistics

Absolute and ln-transformed data are presented in Tables 2 and 3. As expected, all absolute variables showed a skewed distribution. Only the standard deviation of normal-to-normal (NN) intervals (SDNN) standing was normally distributed. Of note, the ln-transformation aimed at normalizing the distribution failed for several variables (Total power (TP), low frequency (LF) normalized units (LFnu), high frequency (HF) normalized units (HFnu) supine, and heart rate (HR), very low frequency (VLF), ratio between LF and HF (LF/HF), LFnu standing). Logically, distinct physiological responses were observed between supine and standing positions suggesting substantial postural influences on autonomic balance^26^. Our HRV values are consistent with established norms for healthy, active individuals^32^. Environmental differences between at-home and in-lab settings also impacted HRV readings, suggesting that the measurement environment plays a substantial role in autonomic activity (see repeated measures analysis below).

**Table 2:** Descriptive statistics for absolute HRV metrics across different measurement points and conditions. Data are presented either in mean ± standard deviations or median [25th;75th percentile]. SDNN (standard deviation of NN intervals), HR (heart rate), RMSSD (root mean square of successive differencess), PNN50% (percentage of successive NN intervals that Ddffer by more than 50 ms), VLF (very low frequency), LF l(ow frequency), HF (high frequency), TP (total power), LFnu (low frequency normalized units), HFnu (high frequency normalized units).

	Measure A	Measure B	Measure C	Measure D	Measure E
*Supine*					
SDNN (ms)	63.8 [47.6;82.4]	60 [42;86.5]	66.6 [45.8;94.3]	62 [51.7;84.8]	57.9 [47.2;75.4]
HR (bpm)	54.7 [50.7;61.1]	57.7 [52.3;63.2]	55 [50.4;60.8]	55.7 [51;63.8]	59.1 [54;67.6]
RMSSD (ms)	51.3 [33.2;81]	56.7 [35.4;88.3]	58.3 [34.7;107]	50.5 [35.6;86.5]	48.7 [31.2;74.2]
PNN50%	29.5 [9.5;53.2]	33 [12.1;60.1]	38.8 [13.4;62.7]	27.4 [16.8;50.6]	29.2 [8.7;51.7]
VLF (ms^2^)	1067.3 [507.9;2423]	515.4 [292.5;1462.9]	1141.4 [466.2;1525.5]	1066.6 [639.2;2505.8]	907.1 [585.4;1538.8]
LF (ms^2^)	1047.2 [468.1;2039.4]	1044 [409;3403.7]	1066.4 [466.9;2150.3]	952.3 [602.4;2302.5]	818.7 [462;1419.3]
HF (m^s2^)	793.6 [389.6;1620.2]	965.9 [471.8;2731.8]	834.3 [500.9;2938.2]	856.5 [379.4;1881.8]	869.3 [502.4;1637.7]
LF/HF	1.1 [0.6;2.5]	1.1 [0.5;3.1]	1 [0.5;2.1]	1.4 [0.7;2.3]	0.9 [0.5;3.2]
LF + HF (ms^2^)	1980.6 [1149.5;4154.6]	3042.1 [1273.9;5908.7]	2551.8 [968.2;6088.9]	2396.6 [1177.6;4041.1]	1970.3 [975.9;3574]
TP (ms^2^)	2684 [2092.2;6406.1]	3627.7 [1642.4;6840.4]	4090.1 [1908.5;9417.2]	4014.4 [2099.3;6031.3]	3130.9 [1795.5;5818.6]
HF/HR	13.6 [7;27.9]	17.2 [6.8;52]	12.7 [7.4;55.8]	13.6 [6.5;31.7]	15 [6.4;33.6]
LF + HF/HR	32.4 [21.7;76.4]	39.7 [19.6;103.6]	44.1 [14.5;129.5]	37.7 [21;73.7]	34.9 [15.7;63.3]
LFnu	53.5 [36.4;71.1]	53.1 [32;74.2]	48.7 [32.2;67.5]	58.3 [40.7;69.8]	46.9 [31.4;75.9]
HFnu	46.5 [28.9;63.6]	46.9 [25.8;68]	51.3 [32.5;67.8]	41.7 [30.2;59.3]	53.1 [24.1;68.6]
*Standing*					
SDNN (ms)	55.3 ± 25	44.4 ± 19.2	50.8 ± 22.4	49.5 ± 18.5	43.4 ± 17.7
HR (bpm)	80 [77;93]	77.9 [69.2;84.3]	79.4 [70.4;83.5]	86.4 [76.1;91.2]	80.2 [71.5;88.6]
RMSSD (ms)	19.4 [13.5;25]	17.1 [13.6;25.5]	19.5 [14.5;29.6]	15.8 [11.3;25.2]	17.4 [12.4;23.6]
PNN50%	2.3 [0.3;4.3]	0.9 [0;5.3]	2.4 [0.3;7.4]	1.4 [0.1;4.7]	1.5 [0;4.1]
VLF (ms^2^)	833.3 [445;1729.3]	585 [329;1137.5]	869.4 [268.1;1216.5]	740 [515.5;1539.6]	435 [259.8;869]
LF (ms^2^)	1433.2 [508.4;2190.6]	713.6 [238.2;1123.7]	661.9 [342.5;1773.6]	1076.8 [515.6;2478.4]	507.7 [292.3;1065.3]
HF (ms^2^)	116.7 [62.5;226.2]	137 [62.9;207.6]	160.3 [101.4;263.3]	85.7 [45.9;173.1]	103.2 [78.9;230.9]
LF/HF	11.2 [5.9;17.7]	5.3 [2.1;12.7]	3.7 [2.7;9.8]	13.6 [8.3;20]	4.8 [3;7.6]
LF + HF (ms^2^)	1483 [637.4;2445]	1028.8 [356.2;1258.2]	906.6 [498;2036.4]	1184.1 [613.6;2611.5]	659.9 [355;1310.1]
TP (ms^2^)	2765 [1166.9;4423.7]	1422.9 [773;2548.4]	1970.4 [997.3;3450.7]	2537.9 [1162.2;3899.3]	1196.1 [751.1;2469.6]
LF/HR	17.5 [6.2;28]	9 [3.3;17.4]	9.1 [3.7;21.7]	13.1 [6.3;30.2]	6.9 [3.4;13.6]
LFnu	91.8 [85.4;94.7]	84 [67.4;92.7]	78.7 [72.7;90.7]	93.2 [89.3;95.2]	82.7 [74.7;88.4]
HFnu	8.2 [5.3;14.6]	16 [7.3;32.6]	21.3 [9.3;27.3]	6.8 [4.8;10.7]	17.3 [11.6;25.3]

**Table 3:** Descriptive statistics for ln-transformed HRV metrics across different measurement points and conditions. Data are presented either in mean ± standard deviations or median [25th;75th percentile]. SDNN (standard deviation of NN intervals), HR (heart rate), RMSSD (root mean square of successive differences), PNN50% (percentage of successive NN intervals that differ by more than 50 ms), VLF (very low frequency), LF (low frequency), HF (high frequency), TP (total power), LFnu (low frequencyy normalized units), HFnu (high frequency normalized units).

	Measure A	Measure B	Measure C	Measure D	Measure E
*Supine*					
SDNN (ms)	4.18 ± 0.48	4.11 ± 0.51	4.19 ± 0.48	4.19 ± 0.39	4.07 ± 0.45
HR (bpm)	4.04 ± 0.15	4.08 ± 0.19	4.02 ± 0.17	4.03 ± 0.15	4.09 ± 0.19
RMSSD (ms)	3.95 ± 0.56	3.98 ± 0.67	4.04 ± 0.66	3.97 ± 0.54	3.85 ± 0.7
PNN50%	N/A	N/A	N/A	N/A	N/A
VLF (ms^2^)	7.06 ± 1.09	6.48 ± 1.1	6.87 ± 1.02	7.1 ± 0.87	6.76 ± 1.07
LF (ms^2^)	6.87 ± 1.32	6.98 ± 1.39	6.92 ± 1.21	7.09 ± 1.02	6.72 ± 1.14
HF (ms^2^)	6.72 ± 1.14	6.82 ± 1.3	6.94 ± 1.33	6.83 ± 1.04	6.59 ± 1.5
LF/HF	0.15 ± 0.99	0.16 ± 1.2	-0.02 ± 1.14	0.26 ± 1	0.13 ± 1.24
LF + HF (ms^2^)	7.6 ± 1.16	7.75 ± 1.24	7.77 ± 1.2	7.77 ± 0.91	7.52 ± 1.19
TP (ms^2^)	7.89 [7.65;8.76]	8.2 [7.4;8.83]	8.32 [7.55;9.15]	8.3 [7.65;8.7]	8.05 [7.49;8.67]
HF/HR	2.68 ± 1.18	2.75 ± 1.36	2.92 ± 1.38	2.8 ± 1.11	2.5 ± 1.58
LF + HF/HR	3.56 ± 1.2	3.67 ± 1.31	3.74 ± 1.27	3.74 ± 0.98	3.43 ± 1.28
LFnu	3.98 [3.6;4.26]	3.97 [3.47;4.3]	3.89 [3.47;4.21]	4.07 [3.71;4.25]	3.85 [3.45;4.33]
HFnu	3.84 [3.36;4.15]	3.85 [3.22;4.22]	3.94 [3.48;4.22]	3.73 [3.41;4.08]	3.97 [3.18;4.23]
*Standing*					
SDNN (ms)	3.91 ± 0.48	3.7 ± 0.47	3.83 ± 0.47	3.82 ± 0.42	3.69 ± 0.4
HR (bpm)	4.38 [4.34;4.53]	4.36 [4.24;4.43]	4.37 [4.25;4.42]	4.46 [4.33;4.51]	4.38 [4.27;4.48]
RMSSD (ms)	2.86 ± 0.64	2.88 ± 0.56	3.01 ± 0.55	2.79 ± 0.57	2.86 ± 0.52
PNN50%	N/A	N/A	N/A	N/A	N/A
VLF (ms^2^)	6.73 [6.1;7.45]	6.37 [5.8;7.03]	6.77 [5.59;7.1]	6.61 [6.24;7.34]	6.08 [5.56;6.77]
LF (ms^2^)	7.04 ± 1.23	6.41 ± 1.19	6.6 ± 1.13	6.99 ± 1.07	6.43 ± 1.11
HF (ms2)	4.68 ± 1.12	4.84 ± 1.1	5.03 ± 0.97	4.54 ± 1.15	4.84 ± 0.94
LF/HF	2.41 [1.77;2.88]	1.66 [0.72;2.54]	1.3 [0.98;2.28]	2.61 [2.12;3]	1.56 [1.09;2.03]
LF + HF (ms^2^)	7.15 ± 1.2	6.68 ± 1.1	6.85 ± 1.04	7.11 ± 1.05	6.66 ± 1.04
TP (ms^2^)	7.77 ± 1.01	7.28 ± 1.02	7.52 ± 1	7.7 ± 0.92	7.23 ± 0.87
LF/HR	2.61 ± 1.32	2.05 ± 1.25	2.25 ± 1.22	2.55 ± 1.13	2.05 ± 1.19
LFnu	4.52 [4.45;4.55]	4.43 [4.21;4.53]	4.37 [4.29;4.51]	4.53 [4.49;4.56]	4.41 [4.31;4.48]
HFnu	2.13 ± 0.74	2.76 ± 0.86	2.78 ± 0.8	2.04 ± 0.79	2.79 ± 0.67

### Repeated measures analysis

For absolute data supine, only VLF showed significant differences between sessions (A vs. B (*p* = 0.012) and D vs. E (*p* = 0.022)). Standing, HR, LF, LF/HF, LF + HF, TP, LF/HR, LFnu and HFnu showed significant differences exclusively on home vs. lab analysis (*p* = 0.016 ,0.0001, 0.0005, 0.019, 0.035, 0.003, 0.0006, 0.0006, respectively). No differences were observed between daily successive measurements (24-h) performed at-home (A vs. D) and in-lab (B vs. E). For ln-transformed data supine, LF, LF + HF and LF + HF/HR (*p* = 0.041, 0.037, 0.032, respectively) showed significant interaction effects but multiple comparisons analysis did not allow for decisive separation between sessions, with only trends observed for B vs. E (in-lab 24 h, *p* = 0.0113, 0.09 and 0.078, respectively). Standing, HR, LF/HF, LFnu showed post-hoc significant differences with HR being lower in-lab (A vs. B, *p* = 0.016) and LF/HF and LFnu being lower in-lab (*p* = 0.0005 and 0.001 for A vs. B and D vs. E). To evaluate short-term physiological responses within the lab, paired comparisons between measurements B and C showed significant difference for absolute HR supine (+ 2.7 bpm for B; *p* < 0.0001). Supine root mean square of successive differences (RMSSD) was higher for C (*p* = 0.041). Standing, SDNN, RMSSD and VLF were higher for C (*p* = 0.012, 0.021, 0.034, respectively). ANOVA results are available in supplementary materials.

### Relative reliability analysis

The exhaustive results of the relative reliability analysis are presented in Tables 4 and 5. The intraclass correlation coefficients (ICCs) across the five different sessions demonstrated varying degrees of reliability. Both absolute and ln-transformed data showed comparable reliability, with slightly higher values for ln-transformed data in the frequency-domain metrics.

**Table 4:** Intraclass correlation coefficients (ICCs) with 95% confidence intervals for heart rate variability (HRV) absolute metrics across various measurement conditions. SDNN (standard deviation of NN intervals), HR (heart rate), RMSSD (root mean square of successive differences), PNN50% (percentage of successive NN intervals that differ by more than 50 ms), VLF (very low frequency), LF (low frequency), HF (high frequency), TP (total power), LFnu (low frequency normalized units), HFnu (high frequency normalized units). Symbols indicate whether the variable differs significantly between the compared sessions. * (*p* < 0.05), ** (*p* < 0.01), *** (*p* < 0.001).

	5 measurements	A vs. B	A vs. D	B vs. C	B vs. E
*Supine*					
SDNN (ms)	0.816 (0.723–0.891)	0.842 (0.708–0.917)	0.846 (0.713–0.92)	0.875 (0.766–0.936)	0.8 (0.638–0.895)
HR (bpm)	0.836 (0.739–0.906)	0.837 (0.668–0.919)	0.884 (0.78–0.94)	0.913 (0.542–0.971)***	0.856 (0.732–0.925)
RMSSD (ms)	0.841 (0.757–0.907)	0.856 (0.733–0.925)	0.917 (0.84–0.958)	0.904 (0.817–0.951)*	0.817 (0.664–0.905)
PNN50%	0.833 (0.747–0.902)	0.811 (0.657–0.901)	0.834 (0.692–0.914)	0.942 (0.887–0.97)	0.863 (0.744–0.929)
VLF (ms^2^)	0.486 (0.332–0.651)	0.39 (0.0803–0.635)*	0.547 (0.262–0.745)	0.476 (0.179–0.696)	0.459 (0.149–0.688)*
LF (ms^2^)	0.778 (0.672–0.866)	0.781 (0.607–0.884)	0.864 (0.744–0.93)	0.822 (0.675–0.907)	0.683 (0.445–0.829)
HF (ms^2^)	0.644 (0.505–0.774)	0.807 (0.648–0.898)	0.792 (0.623–0.89)	0.72 (0.511–0.849)	0.653 (0.405–0.81)
LF/HF	0.483 (0.329–0.649)	0.502 (0.209–0.714)	0.255 (-0.0899-0.544)	0.588 (0.322–0.769)	0.673 (0.436–0.823)
LF + HF (ms^2^)	0.721 (0.598–0.828)	0.827 (0.681–0.91)	0.866 (0.749–0.931)	0.779 (0.601–0.884)	0.605 (0.345–0.78)
TP (ms^2^)	0.735 (0.616–0.838)	0.789 (0.617–0.888)	0.826 (0.679–0.909)	0.781 (0.607–0.884)	0.604 (0.34–0.78)
HF/HR	0.663 (0.527–0.788)	0.841 (0.707–0.917)	0.793 (0.624–0.891)	0.696 (0.474–0.835)	0.657 (0.412–0.813)
LF + HF/HR	0.726 (0.604–0.831)	0.832 (0.692–0.912)	0.874 (0.763–0.935)	0.775 (0.595–0.881)	0.605 (0.345–0.78)
LFnu	0.649 (0.51–0.778)	0.537 (0.244–0.739)	0.567 (0.288–0.758)	0.802 (0.642–0.896)	0.683 (0.451–0.828)
HFnu	0.649 (0.51–0.778)	0.537 (0.244–0.739)	0.567 (0.288–0.758)	0.802 (0.642–0.896)	0.683 (0.451–0.828)
*Standing*					
SDNN (ms)	0.659 (0.51–0.792)	0.67 (0.276–0.849)	0.694 (0.453–0.84)	0.757 (0.516–0.881)*	0.637 (0.368–0.807)
HR (bpm)	0.788 (0.655–0.882)	0.77 (0.45–0.897)*	0.851 (0.714–0.925)	0.96 (0.917–0.981)	0.877 (0.761–0.938)
RMSSD (ms)	0.656 (0.512–0.788)	0.694 (0.453–0.84)	0.749 (0.546–0.87)	0.777 (0.577–0.887)*	0.453 (0.117–0.695)
PNN50%	0.539 (0.379–0.701)	0.481 (0.154–0.712)	0.768 (0.575–0.881)	0.637 (0.374–0.806)*	0.143 (-0.23-0.474)
VLF (ms^2^)	0.248 (0.104–0.44)	0.246 (-0.11-0.547)	0.287 (-0.0616-0.576)	0.232 (-0.133-0.54)*	0.124 (-0.231-0.452)
LF (ms^2^)	0.589 (0.433–0.74)	0.776 (0.434–0.904)***	0.691 (0.451–0.838)	0.571 (0.273–0.768)	0.375 (0.0214–0.643)
HF (ms^2^)	0.638 (0.49–0.776)	0.752 (0.55–0.872)	0.694 (0.454–0.84)	0.877 (0.76–0.938)	0.229 (-0.139-0.538)
LF/HF	0.498 (0.311–0.68)	0.537 (0.126–0.77)***	0.822 (0.666–0.91)	0.681 (0.432–0.833)	0.707 (0.478–0.847)
LF + HF (ms^2^)	0.59 (0.436–0.741)	0.778 (0.492–0.899)*	0.682 (0.437–0.833)	0.597 (0.309–0.783)	0.344 (-0.0147-0.621)
TP (ms^2^)	0.498 (0.337–0.669)	0.684 (0.423–0.837)*	0.556 (0.258–0.757)	0.437 (0.0995–0.683)	0.267 (-0.0957-0.565)
LF/HR	0.578 (0.423–0.731)	0.749 (0.453–0.883)**	0.687 (0.446–0.836)	0.552 (0.249–0.756)	0.333 (-0.0277-0.614)
LFnu	0.442 (0.258–0.632)	0.306 (-0.043-0.594)***	0.615 (0.335–0.795)	0.704 (0.469–0.846)	0.595 (0.313–0.781)
HFnu	0.442 (0.258–0.632)	0.306 (-0.043-0.594)***	0.615 (0.335–0.795)	0.704 (0.469–0.846)	0.595 (0.313–0.781)

**Table 5:** Intraclass correlation coefficients (ICCs) with 95% confidence intervals for heart rate variability (HRV) ln-transformed metrics across various measurement conditions. SDNN (standard deviation of NN intervals), HR (heart rate), RMSSD (root mean square of successive differences), PNN50% (percentage of successive NN intervals that differ by more than 50 ms), VLF (very low frequency), LF (low frequency), HF (high frequency), TP (total power), LFnu (low frequency normalized units), HFnu (high frequency normalized units). Symbols indicate whether the variable differs significantly between the compared sessions. * (*p* < 0.05), ** (*p* < 0.01), *** (*p* < 0.001).

	5 measurements	A vs. B	A vs. D	B vs. C	B vs. E
*Supine*					
SDNN (ms)	0.823 (0.733–0.896)	0.856 (0.733–0.925)	0.807 (0.648–0.899)	0.875 (0.762–0.936)	0.861 (0.741–0.928)
HR (bpm)	0.845 (0.753–0.911)	0.843 (0.689–0.921)	0.885 (0.783–0.941)	0.92 (0.547–0.974)***	0.852 (0.726–0.923)
RMSSD (ms)	0.817 (0.724–0.891)	0.846 (0.713–0.92)	0.847 (0.715–0.92)	0.934 (0.871–0.967)	0.852 (0.718–0.924)
*PNN50%*					
VLF (ms^2^)	0.505 (0.35–0.667)	0.47 (0.139–0.701)	0.514 (0.213–0.725)	0.477 (0.179–0.697)*	0.531 (0.248–0.733)
LF (ms^2^)	0.763 (0.652–0.856)	0.802 (0.641–0.896)	0.639 (0.393–0.801)	0.858 (0.734–0.926)	0.728 (0.522–0.854)
HF (ms^2^)	0.743 (0.626–0.843)	0.83 (0.688–0.911)	0.69 (0.464–0.832)	0.848 (0.719–0.921)	0.815 (0.661–0.903)
LF/HF	0.652 (0.514–0.78)	0.561 (0.275–0.754)	0.54 (0.252–0.741)	0.797 (0.633–0.893)	0.715 (0.498–0.847)
LF + HF (ms^2^)	0.788 (0.686–0.873)	0.877 (0.768–0.936)	0.697 (0.478–0.836)	0.875 (0.764–0.936)	0.793 (0.623–0.891)
TP (ms^2^)	0.777 (0.671–0.866)	0.849 (0.718–0.922)	0.683 (0.455–0.827)	0.819 (0.67–0.905)	0.812 (0.657–0.901)
HF/HR	0.746 (0.63–0.845)	0.819 (0.668–0.905)	0.7 (0.479–0.838)	0.849 (0.721–0.921)	0.814 (0.659–0.902)
LF + HF/HR	0.796 (0.696–0.878)	0.87 (0.758–0.933)	0.714 (0.502–0.845)	0.88 (0.774–0.938)	0.805 (0.643–0.898)
LFnu	0.654 (0.517–0.781)	0.502 (0.199–0.717)	0.576 (0.299–0.763)	0.792 (0.626–0.89)	0.685 (0.454–0.83)
HFnu	0.617 (0.473-0.755)	0.582 (0.307-0.767)	0.446 (0.13-0.679)	0.764 (0.58-0.874)	0.721 (0.508-0.851)
*Standing*					
SDNN (ms)	0.732 (0.599–0.842)	0.725 (0.33–0.88)	0.764 (0.567–0.879)	0.802 (0.587–0.905)*	0.749 (0.54–0.871)
HR (bpm)	0.774 (0.634–0.874)	0.746 (0.406–0.886)*	0.854 (0.721–0.927)	0.959 (0.916–0.98)	0.864 (0.738–0.932)
RMSSD (ms)	0.717 (0.586–0.83)	0.706 (0.471–0.847)	0.785 (0.602–0.89)	0.848 (0.682–0.927)*	0.671 (0.418–0.827)
*PNN50%*					
VLF (ms^2^)	0.366 (0.21–0.554)	0.525 (0.206–0.741)	0.599 (0.319–0.784)	0.609 (0.335–0.789)	0.309 (-0.0402-0.593)
LF (ms^2^)	0.733 (0.587–0.847)	0.764 (0.16–0.916)	0.738 (0.523–0.865)	0.805 (0.636–0.901)	0.744 (0.531–0.868)
HF (ms^2^)	0.549 (0.391–0.709)	0.513 (0.2-0.731)	0.584 (0.295–0.775)	0.721 (0.501–0.855)	0.399 (0.0496–0.659)
LF/HF	0.5 (0.29–0.69)	0.479 (-0.0235-0.757)***	0.703 (0.47–0.844)	0.755 (0.549–0.874)	0.664 (0.407–0.823)
LF + HF (ms^2^)	0.731 (0.597–0.841)	0.757 (0.395–0.895)	0.723 (0.498–0.856)	0.803 (0.633-0.9)	0.691 (0.448–0.839)
TP (ms^2^)	0.685 (0.537–0.812)	0.701 (0.299–0.867)	0.733 (0.515–0.862)	0.779 (0.582–0.888)	0.641 (0.373–0.81)
LF/HR	0.756 (0.626–0.859)	0.788 (0.333–0.918)	0.757 (0.553–0.875)	0.82 (0.659–0.909)	0.755 (0.549–0.874)
LFnu	0.41 (0.237-0.6)	0.235 (-0.0685-0.52)***	0.593 (0.303–0.781)	0.674 (0.423–0.828)	0.548 (0.252–0.752)
HFnu	0.509 (0.295–0.698)	0.52 (-0.0184-0.788)	0.715 (0.489–0.851)	0.762 (0.56–0.878)	0.678 (0.429–0.831)

In the supine position, time-domain variables such as HR, SDNN and RMSSD exhibited consistently good ICCs (> 0.75), indicative of robust autonomic signal detection, irrespective of transformation. Conversely, frequency-domain metrics displayed moderate reliability (ICCs < 0.75), suggesting some sensitivity to testing conditions, except for LF which showed good reliability (ICCs > 0.75). In the standing position, time-domain metrics and frequency-domain metrics LF and HF demonstrated moderate reliability (ICCs < 0.75), generally lower than in the supine position. Only HR, lnHR, and lnLF/HR reached good reliability, emphasizing the impact of postural changes on measurement stability.

Day-to-day analysis revealed variability in reliability. Supine absolute HR, RMSSD, LF, LF + HF, LF + HF/HR demonstrated good reliability across at-home measurements, while ln-transformed data showed lower ICCs in comparison. In-lab reliability (B vs. E) was significantly lower, especially for frequency-domain analysis. Supine RMSSD consistently exhibited good to excellent reliability across all testing conditions. In contrast, the LF/HF ratio exhibited moderate to poor reliability in certain conditions. For standing measurements, HR exhibited excellent reliability for in-lab comparisons (B vs. C). Most metrics in the standing position demonstrated moderate reliability (ICCs < 0.75), indicating reduced reliability compared to other metrics and conditions. This finding underscores the potential challenges of capturing reliable frequency-domain data in upright postures over extended intervals. Short-term in-lab (A vs. B) measurements showed good to excellent reliability in most of the variables used for clinical interpretation^25^ (e.g. HR, LF, HF), with ln-transformation trending to increase reliability.

### Absolute reliability analysis

Table 6 presents absolute reliability data for all situations from absolute variables mainly used for clinical interpretation. In the supine position, Median Absolute Deviation (MAD) for HR ranged from 1.6 bpm to 2.4 bpm, indicating measurements with minor fluctuations. For SDNN and RMSSD, MAD values varied from 6.3 to 7.9 ms, reflecting modest variance. HF exhibited higher fluctuation with MADs up to 519.6 ms^2^, emphasizing the sensitivity of frequency-domain parasympathetic activity markers to measurement conditions. In the standing position, the MAD for HR demonstrated a range from 1.3 bpm to 3.3 bpm. The LF component showed noteworthy variance with MAD extending to 573 ms^2^. Relative Standard Error of Measurement (SEM) and Minimal Detectable Change (MDC) were significantly higher for frequency-domain variables, with ranges from 1.2 to 9.1% for time-domain and from 16.8 to 34.4% for frequency-domain. Conditions with the lowest SEM were at-home (A vs. D) and short-term in-lab (B vs. C).

**Table 6:** Absolute reliability variables according to each situation for main absolute variables of clinical interest. SEM (standard error of measurement), CV (coefficient of variation), MDC (minimally detectable change).

	5 m	A vs. B	A vs. D	B vs. C	B vs. E
*Heart rate supine*					
SEM (bpm)	1.5 ± 0.8	1.4 ± 1	0.8 ± 0.7	0.7 ± 0.6	1.1 ± 1.2
Relative %	2.5 ± 1.2	2.3 ± 1.5	1.3 ± 1.1	1.2 ± 1	1.9 ± 2
CV	6.2 ± 3.1	5.8 ± 3.6	4 ± 3.2	4 ± 3.4	4.9 ± 5.2
Median of absolute deviation (MAD)	2.1 ± 1.7	2.4 ± 1.7	1.6 ± 1.4	1.7 ± 1.5	2.1 ± 2.2
Relative deviation index (RDI) (%)	3.6 ± 2.6	4.1 ± 2.6	2.8 ± 2.2	2.8 ± 2.4	3.5 ± 3.7
MDC absolute (bpm)	4.1 ± 2.2	3.9 ± 2.8	2.1 ± 1.8	1.9 ± 1.8	3.1 ± 3.3
MDC relative (%)	7 ± 3.6	6.4 ± 4.1	3.7 ± 3	3.2 ± 2.8	5.2 ± 5.5
*SDNN supine*					
SEM (ms)	5.4 ± 3	4.3 ± 3.7	3.9 ± 3.5	3.2 ± 2.8	4.6 ± 4.6
Relative %	8 ± 3.6	6.1 ± 4.4	5.7 ± 4.7	4.6 ± 4.2	6.5 ± 4.8
CV	18.6 ± 8.3	15.4 ± 11.1	14.5 ± 12.1	13.1 ± 11.8	14.6 ± 10.8
Median of absolute deviation (MAD)	6.3 ± 5.7	7.6 ± 6.6	7.1 ± 6.2	6.5 ± 5.6	7.2 ± 7.3
Relative deviation index (RDI) (%)	9 ± 5.8	10.9 ± 7.8	10.2 ± 8.6	9.3 ± 8.4	10.4 ± 7.6
MDC absolute (ms)	14.9 ± 8.2	11.8 ± 10.3	10.9 ± 9.6	9 ± 7.8	12.7 ± 12.8
MDC relative (%)	23.1 ± 11.8	16.9 ± 12.2	15.8 ± 13.2	12.9 ± 11.6	18.1 ± 13.4
*RMSSD supine*					
SEM (ms)	5.3 ± 3.4	4 ± 4	2.3 ± 1.9	2.7 ± 3.2	4.8 ± 5.7
Relative %	9.1 ± 5.2	7 ± 5.8	4.5 ± 4.2	4 ± 3.5	7.7 ± 7.9
CV	22.9 ± 13	18.5 ± 15.4	15.4 ± 14.5	13 ± 11.2	18 ± 18.3
Median of absolute deviation (MAD)	7.5 ± 6	7.5 ± 7.3	5.7 ± 4.8	6.1 ± 7.2	7.9 ± 9.4
Relative deviation index (RDI) (%)	12.4 ± 8	13.1 ± 10.9	10.9 ± 10.3	9.2 ± 7.9	12.7 ± 12.9
MDC absolute (ms)	14.8 ± 9.5	11.2 ± 11	6.4 ± 5.4	7.4 ± 8.8	13.3 ± 15.9
MDC relative (%)	27.1 ± 19.6	19.4 ± 16.1	12.4 ± 11.6	11.2 ± 9.6	21.4 ± 21.8
*HF supine*					
SEM (ms^2^)	513.4 ± 578.5	210.3 ± 263.8	215.4 ± 275.8	388.8 ± 560.5	372.4 ± 652.1
Relative %	31.6 ± 15.2	16.8 ± 11.5	17.5 ± 15	19.8 ± 14.3	20.5 ± 18.6
CV	52.9 ± 25.5	38.3 ± 26.2	38.4 ± 33	37.4 ± 27.1	34.8 ± 31.6
Median of absolute deviation (MAD)	464.8 ± 578.5	338.5 ± 424.6	333.9 ± 427.7	519.6 ± 749	447.1 ± 782.7
Relative deviation index (RDI) (%)	31.5 ± 17.8	27.1 ± 18.5	27.2 ± 23.3	26.4 ± 19.1	24.6 ± 22.3
MDC absolute (ms^2^)	1423.1 ± 1603.4	582.9 ± 731.3	597 ± 764.6	1077.7 ± 1553.5	1032.3 ± 1807.4
MDC relative (%)	119.7 ± 149.7	46.6 ± 31.9	48.5 ± 41.7	54.9 ± 39.7	56.9 ± 51.6
*LF + HF/HR supine*					
SEM (a.u.)	15 ± 15.3	8.5 ± 10	6.6 ± 7.4	11.1 ± 14.3	17 ± 23.7
Relative %	23.9 ± 14.1	12.8 ± 11.2	12.3 ± 10.9	15.6 ± 11.9	24.8 ± 17.8
CV	45.7 ± 26.8	31.5 ± 27.2	34.6 ± 30.7	32.7 ± 24.9	39.6 ± 28.2
Median of absolute deviation (MAD)	17 ± 18.4	14.6 ± 17.3	13.2 ± 14.7	16.5 ± 21.4	19.1 ± 26.6
Relative deviation index (RDI) (%)	27.6 ± 15	22.2 ± 19.2	24.5 ± 21.7	23.1 ± 17.6	28 ± 19.9
MDC absolute (a.u.)	41.7 ± 42.5	23.5 ± 27.8	18.3 ± 20.5	30.7 ± 39.8	47.1 ± 65.6
MDC relative (%)	88.5 ± 120.2	35.5 ± 31.1	34.2 ± 30.3	43.2 ± 32.9	68.8 ± 49.3
*HR standing*					
SEM (bpm)	2.5 ± 1.3	2.4 ± 2.3	1.6 ± 1.1	0.4 ± 0.4	1.3 ± 1.1
Relative %	3.1 ± 1.6	2.9 ± 2.7	1.8 ± 1.2	0.5 ± 0.5	1.6 ± 1.3
CV	6.8 ± 3.5	6.1 ± 5.7	4.6 ± 3.2	2.4 ± 2.3	4.6 ± 3.8
Median of absolute deviation (MAD)	3.3 ± 2.2	3.5 ± 3.3	2.9 ± 2	1.3 ± 1.4	2.6 ± 2.1
Relative deviation index (RDI) (%)	4.1 ± 3	4.3 ± 4	3.3 ± 2.3	1.7 ± 1.6	3.3 ± 2.7
MDC absolute (bpm)	7 ± 3.6	6.6 ± 6.3	4.4 ± 3.1	1 ± 1.1	3.5 ± 2.9
MDC relative (%)	8.9 ± 4.8	8.2 ± 7.6	5 ± 3.5	1.3 ± 1.3	4.4 ± 3.7
*LF standing*					
SEM (ms^2^)	535.9 ± 565.1	321.7 ± 397	450.5 ± 511.7	389.1 ± 578.6	506.4 ± 911.5
Relative %	34.4 ± 12.7	21.5 ± 13.4	22.9 ± 15.5	27.3 ± 13.8	32.7 ± 22.8
CV	53.7 ± 19.8	45.5 ± 28.4	41.2 ± 28	41.7 ± 21.1	41.3 ± 28.8
Median of absolute deviation (MAD)	571.3 ± 774.1	480.6 ± 593.2	573 ± 650.9	420.1 ± 624.7	452.9 ± 815.3
Relative deviation index (RDI) (%)	37.8 ± 19.6	32.2 ± 20.1	29.1 ± 19.8	29.5 ± 14.9	29.2 ± 20.4
MDC absolute (ms^2^)	1485.4 ± 1566.3	891.6 ± 1100.5	1248.6 ± 1418.3	1078.6 ± 1603.8	1403.7 ± 2526.6
MDC relative (%)	112.6 ± 64.2	59.6 ± 37.2	63.4 ± 43.1	75.6 ± 38.3	90.5 ± 63.1
*LF/HR standing*					
SEM (a.u.)	7.1 ± 7.8	4.4 ± 5.8	5.8 ± 6.8	5.5 ± 7.8	7.4 ± 13.6
Relative %	34.6 ± 12.4	22.7 ± 14.4	23.5 ± 15.9	28.7 ± 14.4	34.4 ± 24.6
CV	53.1 ± 19.3	44.8 ± 28.5	41.9 ± 28.2	42.3 ± 22	42.2 ± 30.1
Median of absolute deviation (MAD)	7.6 ± 10.1	6.2 ± 8.1	7.3 ± 8.5	5.8 ± 8.3	6.4 ± 11.7
Relative deviation index (RDI) (%)	36.9 ± 20.5	31.7 ± 20.2	29.7 ± 19.9	29.9 ± 15.6	29.9 ± 21.3
MDC absolute (a.u.)	19.7 ± 21.8	12.1 ± 16	15.9 ± 18.8	15.1 ± 21.7	20.4 ± 37.6
MDC relative (%)	113 ± 62.8	62.8 ± 40	65.2 ± 43.9	79.6 ± 39.9	95.2 ± 68.2

Performing the measurement under strictly controlled laboratory conditions (A vs. B and D vs. E) revealed no significant improvement except in supine VLF and standing HR, LF/HF, and LFnu metrics. Conversely, relative reliability was generally superior and SEM inferior when data were collected at home. Our results validate the reliability of short-term heart rate variability measurements under various conditions among healthy, physically active individuals. They confirmed that HRV is a robust and stable biomarker, showing high consistency across different environments—both at home and in laboratory settings—and under varying postural conditions. The observed variations between different postures reinforce the sensitivity of HRV to physiological changes, supporting its broader application in health assessments and performance monitoring.

## Discussion

HRV is a cornerstone measure for evaluating ANS function. Its non-invasive nature and ability to capture subtle fluctuations in cardiac autonomic regulation have spurred significant interest across diverse fields, including sports medicine, telehealth, and chronic disease management. However, debates persist regarding how best to measure and interpret HRV in real-world scenarios. Methodological inconsistencies such as posture, timing, environmental variations challenge the reliability of HRV findings^18,19^. To address these issues, this study aimed to assess the clinical reliability of short-term HRV measurements in supine and standing positions, comparing at-home and in-laboratory settings in healthy active individuals.

### Main findings

Our study contributes to the growing field of HRV research by showing that HRV remains a reliable indicator of autonomic status in young, healthy, active adults, even under varied conditions — including supine and standing postures and at-home and laboratory environments. Indeed, our findings show that short-term HRV measurements, when performed under standardized conditions with rigorous protocols, exhibit good-to-excellent reliability for most of the relevant variables across these conditions. Day-to-day variability was minimal, underscoring the consistency of HRV measurements for monitoring autonomic function in healthy, active individuals. These results reinforce previous work highlighting the robustness of short-term HRV data under carefully controlled conditions^33,34^. Time-domain HRV metrics such as RMSSD and HR were particularly robust across all conditions, with ICCs often exceeding 0.75. Overall, these findings align with the existing literature reporting the reliability of HRV measurements across a variety of physiological states and settings, with intra-class correlation coefficients ranging from 0.69 to 0.90 across multiple sessions^35,36^. Particularly notable are the obtained ICCs for RMSSD (0.917) and for HR (0.88), which are similar or even better to those reported in adolescent athletes (0.71 and 0.88, respectively)^37^. Frequency-domain metrics, while moderately reliable, were more sensitive to variations in posture and environment, in line with previous reports indicating larger standard errors of measurement in certain conditions where controlling all confounding factors is more challenging, such as non-laboratory environments, short sampling intervals, or varying postures^33,34^. The comparison of our data with those obtained in elite athletes – who showed low variance and good reliability of HRV – further corroborates the stability of HRV measurements in strictly controlled conditions^38^. This underscores that HRV’s “signal” can be distinguished from methodological “noise,” contingent on consistent timing, posture control, and careful data handling. Additionally, our findings showed good-to-excellent in-lab short-term reliability particularly in the supine position (B vs. C), confirming previous findings performed across four consecutive 5-minute measurements^39^. This short-term reliability of in-laboratory HRV provides strong support for its use in detecting true changes in cardiac autonomic regulation in pre/post intervention designs^40^. In contrast, the LF/HF ratio demonstrated moderate to poor reliability in certain conditions, underscoring its sensitivity to variations in environmental factors or measurement protocols. This sensitivity may limit its usefulness as a robust autonomic marker, which has already been discussed in the literature, especially in scenarios where consistent data collection across diverse settings is required^41,42^.

Overall, our results highlight the robustness of HRV as a tool for assessing autonomic nervous system function under different conditions and support the use of HRV as a stable biomarker for health and performance monitoring while underscoring the need for rigorous standardization to enhance interpretative accuracy.

### Environmental influence: Home vs. Laboratory

Despite showing no drastic differences between at-home and in-lab environments, home-based measurements showed finer absolute reliability in the supine position. This modest, yet potentially impactful advantage highlights the potential of at-home HRV data collection to provide more consistent conditions compared to in-lab recordings. Measuring HRV upon awakening in the familiar home environment may reduce the influence of incidental physical activity or other confounding factors that may occur when measuring in clinical settings. Indeed, psychological factors such as anxiety and fear may be more likely to arise in unfamiliar environments like laboratories or hospitals^11,43^. These findings align with the growing adoption of remote health monitoring technologies, where wearable sensors and smartphone apps enable continuous physiological tracking without requiring frequent clinical visits^44,45^, benefiting those who cannot easily attend laboratory or clinical appointments—be it due to geographic limitations, mobility constraints, or busy schedules. Notwithstanding this, by carefully controlling confounding factors—such as fasting status and the absence of prior physical activity, we showed that measurements taken in a one-hour slot post-awakening in a laboratory setting can also achieve substantial reliability and offer a robust way of assessing ANS function. This highlights the potential for both approaches to be utilized interchangeably, depending on practical considerations and participant accessibility, without compromising data quality.

### Importance of standardization and remaining challenges

One of the enduring challenges in HRV research is controlling for the myriad of variables that can influence measurements, including circadian rhythms, recent physical activity, emotional stress, and environmental factors such as temperature^12,29,46,47^. In our study, we attempted to minimize these confounders by instructing participants to collect data upon morning awakening, enforcing fasting, and ensuring minimal prior physical activation. These measures aimed to capture a more “baseline” state of autonomic function. Additionally, we rigorously followed the standardized protocols recommended by Schmitt et al. (2015) focusing on a dual-position analysis upon morning awakening to reduce potential confounding factors and maximize consistency^25^. This dual-position protocol also enabled us to explore the influence of posture on HRV, an aspect often overlooked in less standardized protocols^26^. Our approach thus builds on recommended clinically implemented protocols and resonates with broader efforts to unify HRV measurement protocols^15,16,24,27^. Moreover, we applied rigorous standards to our statistical analysis, including adjustments for skewed distributions^48–50^, ensuring that the reliability estimates accurately captured true autonomic function rather than artifacts.

Despite this rigorous design, we acknowledge that several challenges persist. Indeed, variability in personal routines, differences in participants’ sleep patterns, and individual sensitivity to posture shifts may still introduce noise. Moreover, while short-term and day-to-day measurements exhibit good reliability, as indicated by our findings, the literature suggests that long-term HRV tracking (e.g. over months) is more susceptible to within-subject variability^21,51^. Indeed, while our study demonstrated good reliability of HRV measurements over a 24-hour period, longer-term HRV monitoring still faces challenges. Notably, two studies reported substantially lower ICCs for HRV over intervals spanning several months, reflecting considerable within-subject variability and highlighting the limitations of extended HRV monitoring^21,51^. Additionally, factors like aging, lifestyle modifications, or the onset of subclinical conditions may substantially alter baseline autonomic function^12,29^, which highlights the influence of dynamic physiological factors on autonomic regulation over time and underscores the importance of context when interpreting HRV trends over extended timeframes.

### Perspectives – technological advancements

Refining these methodological standards enables future studies to make confident cross-population comparisons and pre-/post-intervention HRV comparisons^29,40^, expanding HRV applications in clinical and non-clinical contexts. Our findings provide a foundation for further investigations in diverse cohorts, from patients with autonomic dysfunction to those aiming to optimize health and performance. Advances in wearable devices and photoplethysmography (PPG) sensor technology are making large-scale, real-time HRV monitoring more feasible, and artifact-correction algorithms promise more convenient HRV data collection in everyday environments. However, while PPG offers convenience, it remains less precise than ECG due to motion artifacts and external factors, with mixed validation results. Time-domain variables like RMSSD show acceptable agreement with ECG^52^, but frequency-domain parameters exhibit lower reliability^53–56^. Thus, while PPG can be suitable for tracking certain isolated metrics or trends, ECG remains the preferred method for clinical applications and research scenarios where high precision is essential. Emerging analytical techniques, including non-linear measures^57^ and artificial intelligence (AI)-driven models, offer new avenues for capturing complex cardiovascular dynamics. AI integration could establish individualized HRV baselines by incorporating various factors (e.g. age, training load, sleep quality, psychosocial stress), and flag deviations to guide training or clinical decisions, thus enhancing personalized healthcare and performance monitoring^58^. Building on our robust reliability data, future studies could leverage AI to deliver real-time feedback, guiding individualized training adjustments or clinical decision-making and thus broadening the impact of HRV monitoring in personalized healthcare.

### Perspectives – clinical applications

The robust HRV reliability shown in our study reinforces the value of HRV as a practical tool in healthcare. The consistency of our measures across multiple days and under varying postural conditions, suggest that clinicians and researchers can use HRV to track autonomic changes in clinical populations with reasonable confidence. In chronic disease management – especially for conditions associated with autonomic dysregulation like hypertension, heart failure, or diabetes – regular HRV assessments could flag early signs of deterioration. Because our results support the feasibility of remote HRV measurements, healthcare providers may integrate home-based data collection to encourage patient engagement and timely intervention. In athletic and sports medicine contexts, the reliable day-to-day readings evidenced in our study suggest that HRV can serve as a trustworthy gauge of training load, recovery status, and overtraining risk. By adapting training programs according to systematic HRV evaluations, coaches and practitioners may optimize athletes’ performance while mitigating injury or burnout^25,48^. Moreover, our findings of stable HRV metrics across different environments support its growing recognition as a psychosomatic indicator – one that could be included in a broader psychophysiological framework for mental health management. The ability to measure HRV consistently in everyday settings lends itself to tracking stress management interventions, therapeutic progress, and overall well-being in populations dealing with anxiety and depression.

### Conclusion

In conclusion, our study confirms HRV as a highly reliable measure of autonomic function in healthy, active adults when assessed under standardized conditions. We observed consistent reliability across postures and environments, highlighting HRV’s adaptability in real-world scenarios. While at-home measurements showed modest yet potentially meaningful advantages for certain metrics, our broader findings underscore HRV’s utility as a stable biomarker for cardiovascular health and training adaptations. Future research integrating wearable sensors and AI analytics could further enhance its diagnostic potential, supporting personalized, data-driven interventions. Moreover, our findings reinforce HRV’s significance as a critical measure in both clinical and athletic settings^7,14,23^. By demonstrating the robustness of HRV measurements, this study underscores the importance of precise and adaptable assessment protocols, ensuring HRV remains a valuable tool in both clinical and athletic contexts.

## Methods

### Study design

This observational study employed a rigorous design to examine the clinical reliability of HRV measurements, notably by contrasting at-home measurements upon awakening with those conducted in a controlled laboratory environment in individuals without any health complaints. After an initial screening visit, participants underwent five supine/standing HRV measurements within a 24-hour period under standardized conditions, as shown in Fig. 1. The first measurement (A) was taken at home upon awakening, followed later that day by two laboratory measurements: one upon arrival (B) and a second after a 15-minute passive sitting break (C). The process was repeated the next morning with another at-home measurement (D) upon awakening and a final lab measurement (E). The study aimed to: (1) assess short-term HRV reliability across these five time points to evaluate consistency; (2) analyze day-to-day variance by comparing HRV measurements taken in different environments across 24-h; (3) determine whether at-home measurements could reliably substitute or complement lab-based assessments; and (4) evaluate short-term reproducibility of lab-based measurements through repeated testing in a controlled setting.

### Participants

Forty-three participants voluntarily took part in the study. Inclusion criteria required participants to be generally healthy, physically active (engaging in more than 150 min of moderate physical activity per week), aged between 18 and 70, free from acute injury or inflammatory disease, and with fatigue level ≤ 14 on the Profile of Mood State questionnaire. Exclusion criteria included pregnancy, current sickness or injury or acute musculoskeletal pathology, current treatment impacting cardiovascular function, current cardiac or pulmonary pathology, recent episodes of loss of balance or unexplained vagal symptoms, loss consciousness within the past year, and/or susceptibility to orthostatic intolerance. This population was chosen due to their stable physiological profiles and the relevance to sports and exercise medicine applications, where accurate monitoring of ANS activity is critical for optimizing health, performance, and recovery. The study was approved by the Canton de Vaud ethics committee (CER-VD, protocol #2020-00071) and conducted in accordance with the ethical standards of the Declaration of Helsinki. All participants signed an informed consent form prior to data collection.

### Heart rate variability

Upon completion of the screening process during the first visit, participants were provided with a heart rate monitor (Polar H10, Polar, Finland) to perform all measurements. They were thoroughly familiarized with the smartphone application (inCORPUS^®^ app, Be.Care, Lausanne, Switzerland) to conduct the HRV measurements at home independently. The principle of the measurement was to record HR at rest in supine and standing positions for 5 min each, as previously described^24^. Given the sensitivity of HRV to various internal and external factors, rigorous instructions were given. External factors (location, time, room temperature, air humidity, noise, devices used, persons present during the measurements) were kept as stable as possible. For all measurements, participants were also instructed to control for internal confounding factors by: (1) avoiding intense physical efforts 48 h before the measurements, (2) abstaining from alcohol 12 h before the measurements, (3) abstaining from nicotine or caffeine after awakening on the day of measurement (4) performing the measurements in a fasting state, free from muscle soreness, illness, and on an empty bladder. Additionally, participants were instructed to relax throughout the duration of the measurement, without focusing their thoughts on anything specific. During the standing phase, participants were asked to keep their arms along the body and remain motionless, without moving their feet, or transferring their weight from one foot to the other. Any interference (movement, coughing, yawning, spasm, etc.) occurring during the recordings had to be reported to the investigators. Laboratory measurements were performed exclusively between 7 and 9 am to minimize the influence of circadian variations, digestion, and other daily stressors. The conditions of measurement were kept identical between visits. Confounding factors were checked at every visit, and measurements that did not meet the required conditions were excluded.

### Data processing and analysis

Analyses were performed on raw RR intervals of the supine and standing phases extracted from the app dashboard. Each file was visually inspected for artifacts and ectopic beats, which were automatically corrected with a dedicated software (Kubios HRV Premium, Kuopio, Finland; automatic beat correction and medium automatic noise detection^59^). Standard time- and frequency-domain variables were calculated automatically by the software on recording periods of 240 s after excluding the first minute^22,60^. Time- and frequency-domain variables retained for analysis were SDNN, reflecting overall HRV^14^, HR, RMSSD, indicative of parasympathetic influence^22^, percentage of successive NN intervals differing by more than 50 ms (pNN50%), VLF, LF, which highlights increased sympathetic activity and baroreflex sensitivity upon standing^61^, HF, which assesses parasympathetic activity and is valuable for evaluating vagal tone under resting conditions, the LF/HF ratio, LF + HF (sum of low and high frequency power), TP, and normalized units such as LFnu and HFnu. Supine, High frequencies and the sum of HF + LF were also calculated relative to HR to provide a comprehensive and normalized view of total autonomic balance, proving to be suitable for clinical assessments as well as for evaluating physiological responses in physically active individuals independent from naturally low breathing rate which could happen in this population^42^. LF in standing position was also transformed to assess the sympathetic nervous system response to orthostatic stress, adjusted for individual heart rate differences.

We performed our analysis on both absolute and ln-transformed HRV data in the same study to both provide direct biological interpretability and meet parametric test assumptions. Normality was tested with Shapiro-Wilk test. For normally distributed variables, parametric tests were used and descriptive statistics are presented as mean ± standard deviation (SD). If normality failed, data are presented as median [25th ;75th percentile].

To investigate significant differences between measurements, we performed a two-way repeated measures (RM) ANOVA focusing on measures A, B, D and E, which allowed the assessment of main and interaction effects of time (Day 1 vs. Day 2) and environment (at-home vs. in-lab). Measure C was excluded from this analysis as it introduced within-day variability that could confound the effects of the environment (at-home vs. in-lab) and across-day changes. For normally distributed data, a RM ANOVA with post-hoc Fisher’s Least Significant Difference test was used to explore specific pairwise comparisons between groups under different conditions. For non-normally distributed data, the Friedman test with post-hoc Dunn’s multiple comparison test was applied.

To evaluate short-term physiological responses within the lab, paired comparisons between measurements B and C were analyzed using paired t-tests or Wilcoxon signed-rank test, depending on data normality.

To assess relative reliability, we computed single measures intraclass correlation coefficients with a two-way random model, single measures, and absolute agreement. Data are presented with 95% confidence. ICCs were calculated over all 5 data points (measurement A to E) as well as between the following recordings: (1) At-home Day 1 (Measure A) vs. In-lab Day 1 (Measure B), (2) At-home Day 1 (Measure A) vs. At-home Day 2 (Measure D), (3) In-lab Day 1 (Measure B) vs. In-lab Day 1 (Measure C), (4) In-lab Day 1 (Measure B) vs. In-lab Day 2 (Measure E). ICCs were interpreted as < 0.5 = “poor”, 0.5–0.75 = “moderate”, 0.75–0.9 = “good”, > 0.9 = “excellent” reliability^62^.

To assess absolute reliability and clinical interpretations, we restricted our analysis to the absolute variables most used in clinical settings: SDNN, HR, RMSSD, HF; LF + HF/HR in the supine phase, and HR; LF, LF/HR in the standing phase. For each of those variables, SEM was calculated using the mean of standard deviations between each paired measurements, multiplied by √(1-ICC)^50^. SEM was then presented in absolute and relative values (SEM/mean values of the measurements). Coefficient of variation (CV) was calculated by dividing standard deviation by the mean of each configuration (e.g. A vs. B). The 95% limits of agreement (LOA) to determine MDC) were calculated as ± 1.96×√2×SEM^49^ for each SEM, and presented both in absolute and relative value (percentage of the medians). Considering the skewed distribution we also utilized the median absolute deviation (MAD) and Relative Deviation Index (RDI). Absolute deviation from the median was calculated from each measure and MAD was calculated by taking the mean of absolute deviations of the medians. The use of MAD offers then a robust assessment of variability less affected by outlier and skewed distribution. The relative deviation index was calculated by expressing this value in function of the median. The use of MAD and RDI ensures that the analyses are both statistically robust and clinically relevant. All analysis were performed on each situation (all 5 measurements, A vs. B, A vs. D, B vs. C and B vs. E.)

Statistical analyses were conducted using several software tools to ensure comprehensive data evaluation: intraclass correlation coefficients were calculated using Jamovi 2.3.28 (The Jamovi Project, Sydney, Australia); normality tests and comparisons analysis were performed with GraphPad Prism 10.1.2 (324) (GraphPad Software, San Diego, CA, USA); standard error of measurement and limits of agreement analysis were facilitated by Microsoft Excel (Microsoft Corporation, Redmond, WA, USA, Version 2402). Statistical significance was established at *p* < 0.05.

## Electronic supplementary material

Below is the link to the electronic supplementary material.

Supplementary Material 1

## Data Availability

The datasheet and the raw data presented in this study are available on request from the corresponding author.

## Abbreaviations

ANS: Autonomic nervous system
AI: Artificial intelligence
CER-VD: Commission cantonale d’éthique de la recherche sur l’être humain
CV: Coefficient of variation
HF: High frequency
HFnu: High frequency normalized units
HR: Heart rate
HRV: Heart rate variability
ICCs: Intraclass correlation coefficients
LF: Low frequency
LF/HF: Ratio between LF and HF
LFnu: Low frequency normalized units
LOA: Limits of agreement
MAD: Median absolute deviation
MDC: Minimal detectable change
NN: Normal-to-normal
PNN50%: Percentage of successive normal-to-normal intervals that differ by more than 50 ms
POMS: Profile of mood states
PPG: Photoplethysmography
RDI: Relative deviation index
RM: Repeated measures
RMSSD: Root mean square of successive differences
SDNN: Standard deviation of normal-to-normal intervals
SD: Standard deviation
SEM: Standard error of measurement
TP: Total power
VLF: Very low frequency
