# Supplementary material for: Assessing the clinical reliability of short-term heart rate variability: insights from controlled dual-environment and dual-position measurements
Source: Sci Rep. 2025 Feb 15;15:5611. doi: 10.1038/s41598-025-89892-3 (PMC11829968; doi:10.1038/s41598-025-89892-3)
Supplement: Supplementary file 1 — Supplementary Material 1 [file 41598_2025_89892_MOESM1_ESM.docx]

# Supplementary materials:

## Table A. Data selection process with exclusion reasons

**
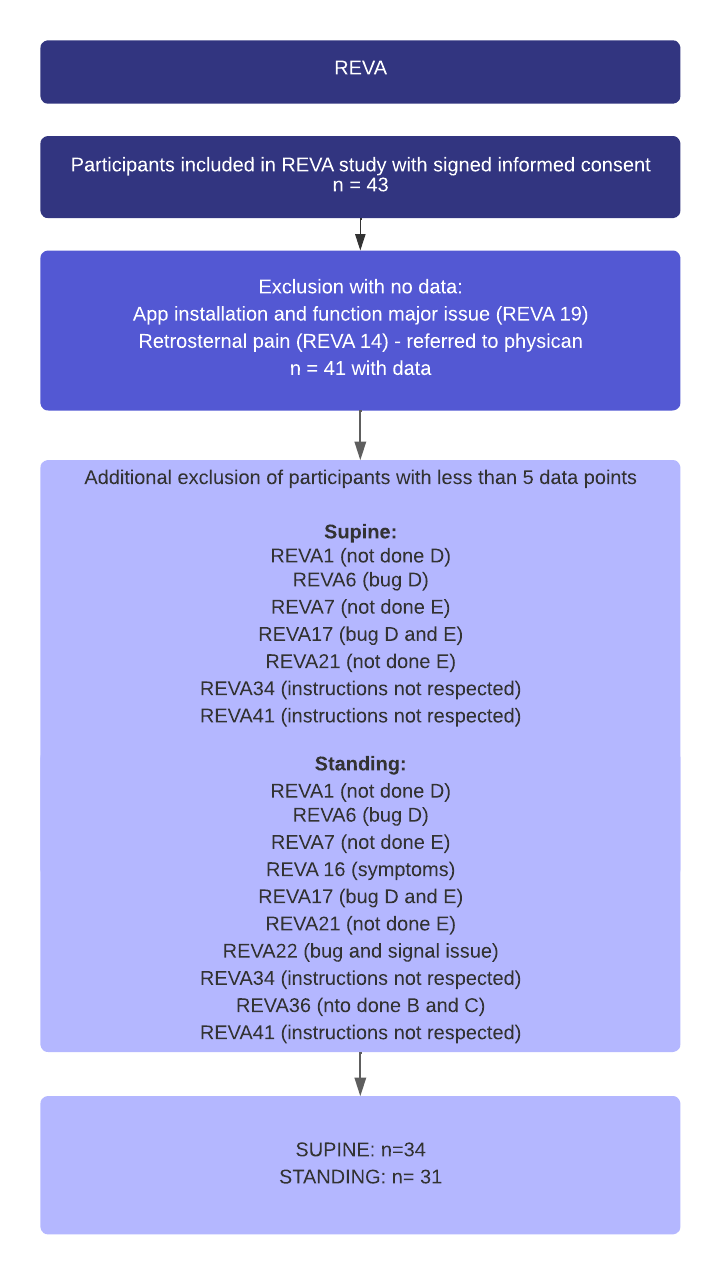
**

## Table B. ANOVA p-values

| Normal distribution - ANOVA | |  |  |  |  |  |  |  |
| --- | --- | --- | --- | --- | --- | --- | --- | --- |
| Non-normal distribution - Friedmann | | | | | | | | |
| Absolute data |  | ANOVA p-value | **A vs B** | **A vs D** | A vs E | B vs D | **B vs E** | D vs E |
| SUPINE | SDNN | 0.0245 | **0.5452** | **>0.9999** | 0.1282 | 0.3249 | **>0.9999** | 0.0672 |
|  | HR | 0.1219 | **0.727** | **>0.9999** | >0.9999 | 0.2327 | **>0.9999** | 0.4935 |
|  | RMSSD | 0.3354 | **>0.9999** | **>0.9999** | >0.9999 | >0.9999 | **0.4458** | >0.9999 |
|  | PNN50% | 0.1605 | **>0.9999** | **>0.9999** | >0.9999 | 0.402 | **0.2327** | >0.9999 |
|  | VLF | 0.0003 | **0.0116** | **>0.9999** | 0.0672 | 0.0031 | **>0.9999** | 0.0216 |
|  | LF | 0.1788 | **>0.9999** | **>0.9999** | 0.7971 | >0.9999 | **>0.9999** | 0.1844 |
|  | HF | 0.5273 | **>0.9999** | **>0.9999** | >0.9999 | >0.9999 | **>0.9999** | >0.9999 |
|  | LF/HF | 0.4356 | **>0.9999** | **>0.9999** | >0.9999 | >0.9999 | **>0.9999** | 0.7971 |
|  | LF+HF | 0.3308 | **>0.9999** | **>0.9999** | >0.9999 | >0.9999 | **0.6618** | 0.7971 |
|  | TP | 0.0858 | **>0.9999** | **>0.9999** | 0.4458 | 0.6618 | **>0.9999** | 0.1131 |
|  | HF/HR | 0.4098 | **>0.9999** | **>0.9999** | >0.9999 | >0.9999 | **>0.9999** | 0.6013 |
|  | LF+HF/HR | 0.3525 | **>0.9999** | **>0.9999** | >0.9999 | >0.9999 | **0.727** | 0.7971 |
|  | Lfnu | 0.5847 | **>0.9999** | **>0.9999** | >0.9999 | >0.9999 | **>0.9999** | >0.9999 |
|  | Hfnu | 0.5847 | **>0.9999** | **>0.9999** | >0.9999 | >0.9999 | **>0.9999** | >0.9999 |
|  |  |  |  |  |  |  |  |  |
| STANDING | SDNN |  |  |  |  |  |  |  |
|  | HR | 0.0024 | **0.0162** | **>0.9999** | 0.0548 | 0.0409 | **>0.9999** | 0.1247 |
|  | RMSSD | 0.7025 | **>0.9999** | **>0.9999** | >0.9999 | >0.9999 | **>0.9999** | >0.9999 |
|  | PNN50% | 0.2972 | **0.9225** | **>0.9999** | 0.6273 | >0.9999 | **>0.9999** | >0.9999 |
|  | VLF | 0.0237 | **0.142** | **>0.9999** | 0.1094 | 0.2948 | **>0.9999** | 0.2331 |
|  | LF | <0.0001 | **0.0001** | **>0.9999** | 0.0001 | 0.0002 | **>0.9999** | 0.0002 |
|  | HF | 0.7763 | **>0.9999** | **>0.9999** | >0.9999 | >0.9999 | **>0.9999** | >0.9999 |
|  | LF/HF | <0.0001 | **0.0005** | **>0.9999** | 0.0049 | <0.0001 | **>0.9999** | 0.0011 |
|  | LF+HF | <0.0001 | **0.019** | **>0.9999** | 0.0007 | 0.026 | **>0.9999** | 0.0011 |
|  | TP | 0.0001 | **0.0353** | **>0.9999** | 0.0007 | 0.0835 | **>0.9999** | 0.0024 |
|  | LF/HR | <0.0001 | **0.0029** | **>0.9999** | 0.002 | 0.0059 | **>0.9999** | 0.0041 |
|  | Lfnu | <0.0001 | **0.0006** | **>0.9999** | 0.0014 | 0.0002 | **>0.9999** | 0.0004 |
|  | Hfnu | <0.0001 | **0.0006** | **>0.9999** | 0.0014 | 0.0002 | **>0.9999** | 0.0004 |
|  |  |  |  |  | HOME | LAB | **DAY1** | DAY2 |
|  |  | Environment x Time | **Environment** | **Time** | DAY1 vs. DAY2 | DAY1 vs. DAY2 | **HOME vs. LAB** | HOME vs. LAB |
|  | SDNN | 0.2437 | **0.0771** | **0.105** | 0.0504 | 0.7416 | **0.036** | 0.2423 |
|  |  |  |  |  |  |  |  |  |
| Ln-transformed data |  |  |  |  | HOME | LAB | **DAY1** | DAY2 |
|  |  | Environment x Time | **Environment** | **Time** | DAY1 vs. DAY2 | DAY1 vs. DAY2 | **HOME vs. LAB** | HOME vs. LAB |
| SUPINE | SDNN | 0.6823 | **0.2405** | **0.8448** | 0.8799 | 0.6688 | **0.5876** | 0.2631 |
|  | HR | 0.311 | **0.2286** | **0.6986** | 0.6564 | 0.3225 | **0.3663** | 0.1523 |
|  | RMSSD | 0.0738 | **0.7416** | **0.2222** | 0.6807 | 0.0347 | **0.8651** |  |
|  | PNN50% | N/A |  |  |  |  |  |  |
|  | VLF | 0.3278 | **0.0363** | **0.185** | 0.8032 | 0.1049 | **0.0204** | 0.1678 |
|  | LF | 0.0407 | **0.6478** | **0.8557** | 0.1827 | 0.1133 | **0.6951** | 0.218 |
|  | HF | 0.1108 | **0.8164** | **0.5781** | 0.4574 | 0.1288 | **0.7431** | 0.4445 |
|  | LF/HF | 0.5117 | **0.8094** | **0.7471** | 0.4891 | 0.813 | **0.9514** | 0.6183 |
|  | LF+HF | 0.0372 | **0.8395** | **0.7587** | 0.2031 | 0.0898 | **0.5859** | 0.3553 |
|  | TP |  |  |  |  |  |  |  |
|  | HF/HR | 0.1043 | **0.7061** | **0.5685** | 0.4503 | 0.1212 | **0.8438** | 0.3642 |
|  | LF+HF/HR | 0.0321 | **0.716** | **0.7321** | 0.1963 | 0.0778 | **0.6973** | 0.2828 |
|  | Lfnu |  |  |  |  |  |  |  |
|  | Hfnu |  |  |  |  |  |  |  |
|  |  |  |  |  |  |  |  |  |
| STANDING | SDNN | 0.2898 | **0.1062** | **0.2275** | 0.111 | 0.9151 | **0.0586** | 0.2453 |
|  | HR |  |  |  |  |  |  |  |
|  | RMSSD | 0.6016 | **0.7488** | **0.3921** | 0.3306 | 0.8124 | **0.9147** | 0.624 |
|  | PNN50% |  |  |  |  |  |  |  |
|  | VLF |  |  |  |  |  |  |  |
|  | LF | 0.7688 | **0.0337** | **0.9195** | 0.78 | 0.8915 | **0.0351** | 0.0576 |
|  | HF | 0.6132 | **0.3412** | **0.6523** | 0.4994 | 0.9689 | **0.5659** | 0.2802 |
|  | LF/HF |  |  |  |  |  |  |  |
|  | LF+HF | 0.926 | **0.0775** | **0.7806** | 0.7928 | 0.8955 | **0.0931** | 0.1077 |
|  | TP | 0.9348 | **0.0367** | **0.5714** | 0.6468 | 0.7318 | **0.0486** | 0.0564 |
|  | LF/HR | 0.8056 | **0.0731** | **0.8077** | 0.7293 | 0.9985 | **0.0753** | 0.108 |
|  | Lfnu |  |  |  |  |  |  |  |
|  | Hfnu | 0.4234 | **0.0002** | **0.6661** | 0.3842 | 0.7933 | **0.0012** | 0.0001 |
|  |  |  |  |  |  |  |  |  |
|  |  | ANOVA p-value | **A vs B** | **A vs D** | A vs E | B vs D | **B vs E** | D vs E |
| SUPINE | TP | 0.0858 | **>0.9999** | **>0.9999** | 0.4458 | 0.6618 | **>0.9999** | 0.1131 |
|  | Lfnu | 0.5847 | **>0.9999** | **>0.9999** | >0.9999 | >0.9999 | **>0.9999** | >0.9999 |
|  | Hfnu | 0.5847 | **>0.9999** | **>0.9999** | >0.9999 | >0.9999 | **>0.9999** | >0.9999 |
|  |  |  |  |  |  |  |  |  |
| STANDING | HR | 0.0024 | **0.0162** | **>0.9999** | 0.0548 | 0.0409 | **>0.9999** | 0.1247 |
|  | VLF | 0.0237 | **0.142** | **>0.9999** | 0.1094 | 0.2948 | **>0.9999** | 0.2331 |
|  | LF/HF | <0.0001 | **0.0005** | **>0.9999** | 0.0049 | <0.0001 | **>0.9999** | 0.0011 |
|  | Lfnu | <0.0001 | **0.0005** | **>0.9999** | 0.0049 | <0.0001 | **>0.9999** | 0.0011 |
